# Xuebijing Injection Alleviates the Inflammatory Response in Patients with Venous-Arterial Extracorporeal Membrane Oxygenation: A Prospective Randomized Controlled Study

**DOI:** 10.31083/j.rcm2511405

**Published:** 2024-11-19

**Authors:** Zhiyong Yuan, Ying Liu, Fuhua Wang, Xiaoning Han, Zhenhui Dong, Jinyan Xing, Xiaotian Chang

**Affiliations:** ^1^Department of Critical Medicine, The Affiliated Hospital of Qingdao University, 266000 Qingdao, Shandong, China; ^2^Medical Research Center of the Affiliated Hospital of Qingdao University, 266000 Qingdao, Shandong, China

**Keywords:** Xuebijing injection, venous-arterial extracorporeal membrane oxygenation, cardiogenic shock, acute myocardial infarction, inflammation storm, cytokine

## Abstract

**Background:**

Both acute myocardial infarction (AMI) and its salvage treatment, venoarterial-extracorporeal membrane oxygenation (VA-ECMO), may lead to the production of proinflammatory cytokines and further aggravate tissue damage. Xuebijing (XBJ) may modulate cytokine production involved in the inflammatory response. We aimed to determine the efficacy of XBJ in cardiogenic shock patients on VA-ECMO.

**Methods:**

This was a prospective, randomized trial carried out in an intensive care unit of a tertiary teaching hospital. Patients with cardiogenic shock after acute myocardial infarction undergoing percutaneous coronary intervention (PCI) with VA-ECMO support were randomly divided into a Xuebijing group and a control group. Cytokines, inflammatory factors and left ventricular ejection fraction (LVEF) were compared between the groups.

**Results:**

41 patients were enrolled in the study, with 21 in the Xuebijing group and 20 in the control group. 28 (68.3%) were male, and the average age was 64.71 ± 8.18 years old. There was no difference in APACHEII (acute physiology and chronic health evaluation II) score, LVEF, or cytokine and inflammatory factors collected before extracorporeal membrane oxygenation (ECMO) between the two groups. The levels of interleukin-6 (IL-6) and tumor necrosis factor-alpha (TNF-α) in the Xuebijing group were lower than those in the control group in the first 24 hours, 48 hours and 72 hours after ECMO (*p* < 0.05). The LVEF in the Xuebijing group was higher than that of the control group at 48 hours (31.57 ± 3.43 *vs*. 28.35 ± 4.42, *p* = 0.013). This trend persisted at 72 hours. The duration of ECMO support in the Xuebijing group was 5.57 ± 2.11 days, which was shorter than that in the control group (*p* = 0.033).

**Conclusions:**

Xuebijing injection can reduce the inflammatory response and improve cardiac function in patients with acute myocardial infarction treated with VA-ECMO to a certain extent.

**Clinical Trial Registration:**

Chinese Clinical Trial Registry (ChiCTR), ChiCTR2100054069, Registered 8, December 2021, https://www.chictr.org.cn/showproj.html?proj=142869.

## 1. Introduction

Venoarterial-extracorporeal membrane oxygenation (VA-ECMO) is a life-saving therapy, which can provide circulatory and respiratory support for patients with severe cardiac and/or respiratory failure [1]. Acute myocardial infarction (AMI) is the most common cause of cardiogenic shock (CS), named acute myocardial infarction with cardiogenic shock (AMICS). It is a class of clinical syndromes in which the cardiac output is significantly reduced due to acute myocardial infarction, resulting in tissue hypoperfusion. It may manifest as recurrent or progressive ischemic symptoms that are difficult to control with drugs, accompanied by hemodynamic instability, life-threatening arrhythmias, cardiac arrest, and acute heart failure [2]. The use of VA-ECMO is becoming increasingly popular in the treatment of cardiogenic shock as a salvage therapy. However, with improvement of extracorporeal membrane oxygenation (ECMO) circuits, advances in catheterization technology and management of ECMO treatment, the survival rate of ECMO has not increased significantly. Less than half of the patients supported with VA-ECMO failed to survive to hospital discharge [1].

In patients with AMI, the expression of cytokines significantly increases both in the infarct and border zone. This is followed by the activation of the complement system, which mediates humoral and cellular responses, leading to further expansion of the inflammatory response [3]. Cardiomyocyte necrosis induces both a systemic response and a local reaction. The recruitment of circulating inflammatory cells in the necrotic area removes dead cells and matrix debris. Cytokines such as interleukin-1 (IL-1), tumor necrosis factor-alpha (TNF-α) and interleukin-6 (IL-6) are produced rapidly in the humoral phase [3]. Early and efficient coronary reperfusion is the main therapeutic goal in AMI. A recent multivariate network meta-analysis confirmed that primary percutaneous coronary intervention (PCI) in a timely manner is the best way to improve clinical outcomes of patients with ST segment elevation myocardial infarction (STEMI), with an odds ratio (OR) of 0.73 (95% confidence interval (CI), 0.61–0.89) when compared with fibrinolytic therapy for mortality [4]. Nevertheless, myocardial ischemia reperfusion induces a sterile inflammatory response that contributes to the final infarct size. Coronary artery diseases, including myocardial infarction (MI) and ischemia/reperfusion injury (IR), account for nearly 50% of heart failure cases [5]. VA-ECMO can provide adequate organ perfusion and temporarily replace cardiopulmonary function. During extracorporeal life support (ECLS), the exposure of the patient’s blood to the artificial, non-endothelialized surfaces of an extracorporeal circuit, leads to increased initiation of coagulation, cellular activation, and increased inflammation, which disrupts the homeostasis of patients who are already seriously ill [6]. During ECMO support, overproduction of pro-inflammatory cytokines leads to capillary leakage, and peripheral and pulmonary edema, which has been found to be a major factor in tissue damage and organ failure [7]. Therefore, reducing the inflammatory response and eliminating pro-inflammatory factors and other chemokines have been approved as effective measures to treat severe sepsis or ECLS-associated systemic inflammatory response syndrome [8].

Xuebijing (XBJ) injection, a drug derived from Chinese herbs, has been approved to treat severe infections including sepsis (China Food and Drug Administration; Beijing, China, Number Z20040033). It is widely used in China and has been used in critically ill patients for more than 10 years. XBJ is composed of five Chinese herbal extracts including Carthami flos, Paeoniae radix rubra, Chuanxiong rhizoma, Salviae miltiorrhizae, and Angelicae Sinensis radix. The dominant components of XBJ that have been monitored include hydroxysafflor yellow A, oxypaeoniflorin, senkyunolide I, and benzoylpaeoniflorin [9]. The incidence and influencing factors of side effects of Xuebijing differ among studies, mainly allergic reaction. Adverse reactions to Xuebijing injections were correlated with vehicle type, dosage, age, and drug combination [10].

It has been demonstrated that XBJ may regulate the production of cytokines, especially pro-inflammatory factors such as TNF-α and IL-6, which play a role in the progression of sepsis. XBJ may play an anti-inflammatory and anticoagulant role in the treatment of sepsis, thereby, regulating the immune response, protecting vascular endothelium and reducing oxidative stress [11, 12]. In a study comparing XBJ with a placebo in critically ill patients with severe community-acquired pneumonia (SCAP), XBJ improved the pneumonia severity index, reduced the duration of mechanical ventilation and length of intensive care unit (ICU) stay, and reduced mortality. This positive result may be attributed to its potential anti-inflammatory and immune-enhancing mechanisms [8].

In clinical studies, there are many anti-inflammatory strategies for ECMO patients, including glucocorticoids, monoclonal antibodies, blood purification technology, but the efficacy remains uncertain [13, 14, 15]. A multi-target approach targeting multiple intracellular signaling pathways may be a more effective strategy for cardiac protection. Accordingly, this prospective, randomized study was conducted to determine the efficacy of XBJ in addition to standard care for cardiogenic shock patients on VA-ECMO.

## 2. Methods and Materials

### 2.1 Design and Setting

This was a prospective, randomized trial carried out in a critical care department of a tertiary teaching hospital, which can independently perform about 100 ECMO cases per year, including more than 70 VA-ECMO cases. It is a single-center, prospective, randomized, double-blinded trial. The trial was approved by Qingdao University Affiliated Hospital (No. qyfykyll 912111920) and registered in the Chinese Clinical Trial Registry (ChiCTR2100054069). Written informed consent was obtained from legally authorized representatives of patients. Enrollment of patients started in December 2021 and ended in December 2022; follow-up finished in June 2023.

### 2.2 Study Population

Patients who met the following criteria were eligible for inclusion: (1) 18–75 years old; (2) diagnosed as AMICS and treated with VA-ECMO.

Patients were excluded if: (1) they had a history of allergy to XBJ; (2) were in a confirmed or suspected immunosuppressive or immunodeficiency state; (3) with known or suspected infection; (4) failure to open a critical stenotic coronary artery vessel; (5) continuous renal replacement therapy (CRRT) was required; (6) acute or chronic hepatic insufficiency.

### 2.3 Randomization and Intervention

Randomization was performed by generating random numbers using a computer, where the XBJ group and the control group were allocated in a 1:1 ratio. The participants received the solvent only (normal saline, 200 mL, q12hr (every 12 hours)) in the placebo group and the solvent plus XBJ (normal saline 100 mL + XBJ 100 mL, q12hr) in the XBJ group. The treatment duration of the study lasted 7 days. All patients received peripheral VA-ECMO catheterization, with the right femoral vein and right femoral artery being preferred, followed by the left femoral vein and left femoral artery. A distal perfusion tube was routinely inserted. Other treatments, including sedative and analgesic management, vasoactive drug administration, ventilator management, and anticoagulant therapy, were performed by the same team according to the guidelines.

### 2.4 Sample Size Calculating

As there are no relevant studies on Xuebijing in acute myocardial infarction, we conducted a prior experiment. 10 patients were randomly assigned to the Xuebijing group or the control group, with 5 in each group, and IL-6 was measured 3 days later. The IL-6 levels in the Xuebijing group and the control group were 50.28 ± 28.34 pg/mL and 75.45 ± 25.24 pg/mL, respectively. The above data was used to calculate the sample size. The distribution between the two groups was 1:1, and the loss to follow-up ratio was 0.1. The samples were about 20 patients in each group.

### 2.5 Data Collection

Data on (1) Patient information: age, sex, body mass index (BMI), comorbidities, time from admission to recanalization (hour), APACHEII (acute physiology and chronic health evaluation II) score, SOFA (Sequential Organ Failure Assessment) score and survival after VA-ECMO (SAVE) score. (2) cytokine and inflammatory factors including IL-6, IL-8, IL-10, TNF-α and C-reactive protein (CRP) before ECMO and 24, 48, 72 hours after administration of ECMO. (3) Coagulation indicators: platelet, D-Dimer and hemoglobin before ECMO and 24, 48, 72 hours after administration of ECMO. (4) Cardiac function was measured using the Simpson method before ECMO and at 24, 48, and 72 hours after ECMO. (5) Patient outcomes: Left ventricular ejection fraction (LVEF) at 28 days after ECMO, length of ICU stay, duration of ECMO, complications including bleeding and thrombosis were recorded.

### 2.6 Statistical Analysis

Normal distribution of all data was checked by the Shapiro-Wilk normality test. Normally distributed data are expressed as the mean and standard deviation. Non-normally distributed data are presented as the median and interquartile range (IQR) and were analyzed using the non-parametric Mann–Whitney U test. Categorical variables are expressed as quantities and percentages and are compared with the chi-square test or Fisher’s exact test. The results are expressed as the *p* value and OR with the 95% CI. *p* < 0.05 is considered as statistically significant. All statistical analyses were performed using IBM SPSS 25.0 software (IBM Corp., Armonk, NY, USA).

## 3. Results

**Fig. 1.**
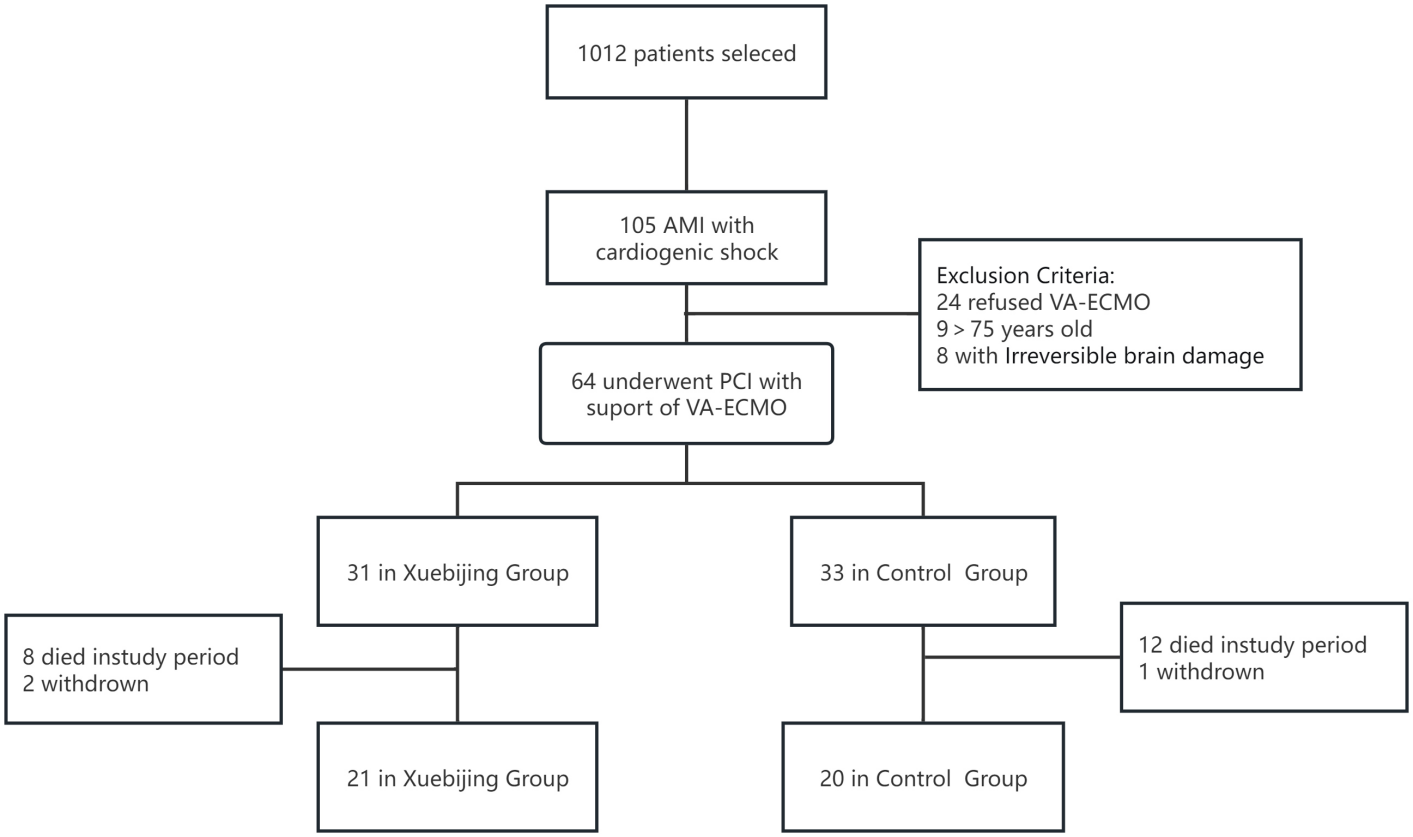
**The flow chart of patient enrollment**. AMI, acute myocardial infarction; VA-ECMO, venoarterial-extracorporeal membrane oxygenation; PCI, percutaneous coronary intervention.

A total of 1012 patients were screened, 105 patients were diagnosed with AMICS, 64 patients selected VA-ECMO treatment, and were randomly divided into the XBJ group and the control group. 41 patients were finally enrolled in the study, 21 in the XBJ group and 20 in the control group. The flow chart of patient enrollment was shown in Fig. 1.

Among the 41 patients, 28 (68.3%) were male, and the average age was 64.71 ± 8.18 years old. The time from onset to blood flow recanalization was 8.37 ± 2.63 hours, and there was no difference in age, gender, comorbidities, APACHEII score, SOFA score or culprit vessels between the two groups. The LVEF was 26.10 ± 6.46% in the XBJ group, and 25.86 ± 5.90% in the control group (*p* = 0.901). The basic characteristics of patients are shown in Table 1.

**Table 1.** **Basic characteristics of patients enrolled**.

		XBJ group	Control group	*p*
		N = 21	N = 20	
Age (year)		64.05 ± 7.90	65.40 ± 8.62	0.603
Sex (male)		14	14	1.000
BMI (kg/m^2^)		24.98 ± 2.02	23.79 ± 2.24	0.082
Co-morbidities				
	Hypertension	14	14	1.000
	Diabetes mellitus	3	6	0.277
	Cerebral infarction	2	4	0.410
	Coronary atherosclerotic heart disease	8	7	0.547
	Peripheral vascular diseases	3	3	0.654
	Chronic pulmonary disease	2	6	0.104
Time from onset to blood flow recanalization (hour)		8.43 ± 2.73	8.30 ± 2.60	0.878
APACHEII score		21.43 ± 4.12	22.20 ± 4.09	0.551
SOFA score		4.52 ± 1.75	4.35 ± 1.73	0.751
SAVE score		–1.10 ± 2.34	–1.15 ± 2.35	0.941
Left ventricular ejection fraction (%)		25.86 ± 5.90	26.10 ± 6.46	0.901
Culprit vessels				
	Right coronary artery	3	3	0.845
	Left main coronary artery	4	3	
	Left anterior descending branch	9	11	
	Left circumflex artery	5	3	

APACHEII score, Acute Physiology and Chronic Health Status score II; SOFA score, Sequential Organ Failure Assessment score; XBJ, Xuebijing; BMI, body mass index; SAVE score, Survival After Veno-arterial Extracorporeal Membrane Oxygenation score. An online calculator is available at https://www.evidencio.com/models/show/1001.

Data on cytokines and inflammatory factors were collected before ECMO and did not differ between the two groups (Table 2). In the first 24 hours, the levels of cytokines were higher than those before ECMO, but the levels of IL-6 and TNF-α in the XBJ group were lower than those in the control group, 171.43 ± 26.68 pg/mL *vs*. 213.20 ± 41.28 pg/mL (*p* = 0.001) and 1.81 ± 0.43 pg/mL *vs*. 2.20 ± 0.53 pg/mL (*p* = 0.014) respectively. There were no differences in other cytokines including IL-8 and IL-10. In the XBJ group, the D-Dimer was lower than that in control group (1055.95 ± 221.73 ng/mL *vs*. 1348.95 ± 230.57 ng/mL, *p* = 0.000), and platelets were higher than that in the control group ((174.10 ± 31.76) × 10^9^/L *vs*. (152.15 ± 35.41) × 10^9^/L, *p* = 0.043). However, there was no difference in CRP levels (Table 3).

**Table 2.** **Inflammatory markers and cardiac function before ECMO**.

	XBJ group	Control group	*p*
	N = 21	N = 20	
Troponin I (ng/mL)	0.91 ± 0.34	0.99 ± 0.46	0.560
Interleukin-6 (pg/mL)	110.14 ± 14.94	109.70 ± 17.45	0.931
Interleukin-10 (pg/mL)	8.84 ± 2.17	8.56 ± 2.04	0.680
Tumor necrosis factor α (pg/mL)	1.21 ± 0.22	1.20 ± 0.23	0.818
Interleukin-8 (pg/mL)	10.23 ± 2.46	10.84 ± 2.51	0.437
Hemoglobin (g/L)	144.48 ± 11.55	140.5 ± 13.77	0.322
Platelet (×10^9^/L)	216.05 ± 36.25	211.45 ± 47.87	0.730
C reactive protein (mg/L)	1.17 ± 0.38	1.34 ± 0.79	0.380
D-Dimer (ng/mL)	286.62 ± 30.61	274.20 ± 52.50	0.365

ECMO, extracorporeal membrane oxygenation; XBJ, Xuebijing.

**Table 3.** **Cytokine and other indicators 24, 48, 72 hours after ECMO**.

		XBJ group	Control group	*p*
		N = 21	N = 20	
24 hours after ECMO				
	Interleukin-6 (pg/mL)	171.43 ± 26.68	213.20 ± 41.28	0.001
	Tumor necrosis factor α (pg/mL)	1.81 ± 0.43	2.20 ± 0.53	0.014
	Interleukin-8 (pg/mL)	4.78 ± 1.30	5.55 ± 1.33	0.071
	Interleukin-10 (pg/mL)	2.03 ± 0.32	2.06 ± 0.38	0.735
	Hemoglobin (g/L)	125.43 ± 12.46	113.25 ± 11.30	0.002
	Platelet (×10^9^/L)	174.10 ± 31.76	152.15 ± 35.41	0.043
	D-Dimer (ng/mL)	1055.95 ± 221.73	1348.95 ± 230.57	0.000
	C reactive protein (mg/L)	78.90 ± 22.05	89.15 ± 18.09	0.113
48 hours after ECMO				
	Interleukin-6 (pg/mL)	75.19 ± 20.63	130.10 ± 27.82	0.000
	Tumor necrosis factor α (pg/mL)	1.13 ± 0.19	1.64 ± 0.49	0.000
	Interleukin-8 (pg/mL)	2.52 ± 1.42	3.11 ± 0.69	0.105
	Interleukin-10 (pg/mL)	1.01 ± 0.20	1.07 ± 0.24	0.343
	Hemoglobin (g/L)	113.90 ± 13.10	100.00 ± 6.94	0.000
	Platelet (×10^9^/L)	135.19 ± 19.91	118.10 ± 18.95	0.008
	D-Dimer (ng/mL)	1388.10 ± 343.66	1968.0 ± 405.28	0.000
	C reactive protein (mg/L)	94.71 ± 12.29	107.70 ± 17.21	0.008
72 hours after ECMO				
	Interleukin-6 (pg/mL)	57.47 ± 11.02	103.70 ± 22.63	0.000
	Tumor necrosis factor α (pg/mL)	1.20 ± 0.20	1.73 ± 0.39	0.000
	Interleukin-8 (pg/mL)	2.46 ± 0.56	3.38 ± 0.70	0.000
	Interleukin-10 (pg/mL)	1.31 ± 0.20	1.27 ± 0.24	0.518
	Hemoglobin (g/L)	98.95 ± 15.49	84.75 ± 6.88	0.001
	Platelet (×10^9^/L)	120.00 ± 22.22	95.80 ± 13.54	0.000
	D-Dimer (ng/mL)	1547.62 ± 426.19	2349.35 ± 528.94	0.000
	C reactive protein (mg/L)	114.19 ± 17.61	131.45 ± 26.03	0.019
Average ECMO flow on day 1 (L/min)		2.85 ± 0.31	2.88 ± 0.39	0.768
Average ECMO flow on day 2 (L/min)		2.88 ± 0.32	2.80 ± 0.24	0.362
Average ECMO flow on day 3 (L/min)		2.14 ± 0.40	2.38 ± 0.33	0.044

ECMO, extracorporeal membrane oxygenation; XBJ, Xuebijing.

After 48 hours, IL-6 and TNF-α levels had further declined. IL-6 and TNF-α in the XBJ group were still lower than those in the control group (75.19 ± 20.63 pg/mL *vs*. 130.10 ± 27.82 pg/mL, *p* = 0.000) and (1.13 ± 0.19 pg/mL *vs*. 1.64 ± 0.49 pg/mL, *p* = 0.000) respectively. Indicators including hemoglobin, platelets, D-dimer and CRP in the XBJ group were lower than those in the control group. The differences in cytokines, inflammatory factors, and coagulation markers persisted after 72 hours (Table 3).

In terms of cardiac function, the difference between the two groups began to appear at 48 hours. The ejection fraction in the XBJ group was higher than that of the control group at 48 hours (31.57 ± 3.43% *vs*. 28.35 ± 4.42%, *p* = 0.013). This trend persisted at 72 hours. However, there was no difference in ejection fraction between the two groups at 28 days of follow-up (42.29 ± 5.38% *vs*. 40.15 ± 4.45%, *p* = 0.175) (Table 4).

**Table 4.** **Left ventricular ejection fraction and outcomes of patients in groups**.

		XBJ group	Control group	*p*
		N = 21	N = 20	
Left ventricular ejection fraction (%)				
	Before ECMO	25.86 ± 5.90	26.10 ± 6.46	0.901
	At 24 hours	28.71 ± 5.18	27.25 ± 5.03	0.364
	At 48 hours	31.57 ± 3.43	28.35 ± 4.42	0.013
	At 72 hours	33.62 ± 3.57	30.70 ± 3.88	0.016
	28 days after ECMO	42.29 ± 5.38	40.15 ± 4.45	0.175
Duration of ECMO (days)		5.57 ± 2.11	7.25 ± 2.73	0.033
Length of stay (days)		12.62 ± 4.78	16.20 ± 8.28	0.096
Bleeding (n)		0	0	
Thrombosis (n)		2	4	0.663
SOFA score at day 28		0 (0, 1)	1 (0, 2)	0.205

ECMO, extracorporeal membrane oxygenation; SOFA score, Sequential Organ Failure Assessment score; XBJ, Xuebijing.

The duration of ECMO in the XBJ group was 5.57 ± 2.11 days, which was shorter than that in the control group (*p* = 0.033). The length of stay in the XBJ group showed a downward trend compared with that in the control group, but was not statistically significant (12.62 ± 4.78 days *vs*. 16.20 ± 8.28 days, *p* = 0.096). During the study period, no bleeding events occurred in the two groups. Two patients in the XBJ group developed embolisms, and 4 in the control group, with no significant difference (Table 4).

## 4. Discussion

The prospective randomized study showed that XBJ could reduce the inflammatory response and improve cardiac function in patients with cardiogenic shock caused by AMI treated with ECMO, but there was no significant difference in cardiac function at 28-day follow-up. To our knowledge, this is the first study of XBJ in patients treated for cardiogenic shock.

AMI remains one of the common causes of hospitalization and death worldwide [16]. Re-perfusion strategies such as thrombolysis and PCI have limited myocardial damage, reduced infarct size, and improved overall prognosis. However, patients with AMI are still confronted with a higher risk of short- and long-term heart failure and even death [17]. The onset of acute myocardial ischemia results in local necrosis, inducing an initial pro-inflammatory response. Circulatory inflammatory cells recruit and remove dead cells and tissues from the MI zone. Myocardial re-perfusion exacerbates proinflammatory response, which is characterized by infiltration of neutrophils resulting in development of necrotic and apoptotic cell death from 6 to 24 hours post-re-perfusion and apoptotic cell death between 48 to 72 hours post-re-perfusion which is associated with large macrophage infiltration and contributes to cardiomyocyte death and oxidative stress [18, 19].

Endothelial dysfunction is more likely to occur in inflammatory states, resulting in increased permeability, subcutaneous accumulation of lipoproteins, leukocyte recruitment, and platelet activation. Macrophages derived from recruited monocytes secrete pro-inflammatory factors, including IL-1β, IL-12, and IL-6, as well as vascular smooth muscle cells [20]. The inflammatory response to AMI plays a critical role in determining MI size, and a persistent pro-inflammatory reaction can contribute to adverse post-MI left ventricular (LV) remodeling [17]. Therefore, the inflammatory response has been a target for cardiac protection.

VA-ECMO is an established treatment for cardiogenic shock, providing both time and perfusion to save heart function. But its complications, whether mechanical, pump-related, or secondary, are common and often lead to morbidity and mortality. After the initiation of ECMO, a rapid rise in pro-inflammatory cytokines, which in severe cases can lead to end-organ dysfunction and death, is thought to be related to the innate immune response [15]. Frerou *et al*. [21] have shown in their prospective study that pro-inflammatory cytokines such as IL-6, IL-8 and TNF-α increased significantly following the initiation of VA-ECMO. The massive activation of inflammatory mediators by the cardiac infarct, which is exacerbated by ECMO, may result in vascular endothelial damage, permeability edema, and impaired oxygen availability to the mitochondria, eventually leading to multiple organ failure [22]. As shown in our study, the levels of inflammatory cytokines at 24 hours after ECMO were increased compared with baseline in both the XBJ and control groups. The DAMPs-TLR4-NF-KB pathway may play an important role in activating inflammation during ECMO. The damage of ECMO catheterization and primary diseases lead to the release of damage-associated molecular patterns (DAMPs), acts on pathogen recognition receptors including toll-like receptor 4 (TLR4), activates nuclear factor kappa-light-chain-enhancer of activated B cells (NF-KB), and promotes the release of inflammatory factors [13].

Xuebijing, a drug used for the treatment of severe community-acquired pneumonia and sepsis, also plays an important role in the treatment of coronavirus disease 2019 (COVID-19) [23]. The multiple active components, multiple targets, and multiple pathways of XBJ injection also provide a new perspective for the study of cardiac shock with ECMO treatment. XBJ has anti-inflammatory and anticoagulation effects, regulates immune responses, protects the vascular endothelium and prevents oxidative stress, which may inhibit the chronic inflammatory response caused by atherosclerosis [24]. Recent studies have shown that XBJ injection can attenuate the excessive production of various inflammatory mediators such as IL-6, TNF-α, monocyte chemoattractant protein-1 (MCP-1), macrophage inflammatory protein-2 (MIP-2), and IL-10 in serum [11, 23]. This is consistent with our findings that levels of IL-6 and TNF-α were decreased in the XBJ group after 48 hours of ECMO.

Although the levels of cytokines decreased in the XBJ group, there was no effect on cardiac function at 28-day follow-up. This may result from ventricular remodeling due to a persistent chronic inflammatory state, basic heart function and the patients’ condition.

## 5. Limitation

This study has several limitations that have to be acknowledged. Although this was a prospective randomized controlled study, blinding was difficult due to the characteristics of XBJ. The strict inclusion and exclusion criteria meant that only few patients could be included. Secondly, as there are no relevant studies on Xuebijing in AMI, the sample size was calculated from our prior experiment. Only 41 patients have been included over a year period. The small size of both groups undoubtedly limits the value of our results. Lastly, the outcome of patients is greatly affected by the experience of ECMO center, which may vary among centers. Therefore, larger multicenter trials are needed to confirm the reliability and effectiveness of XBJ.

## 6. Conclusions

Although no long-term improvement in cardiac function was shown, Xuebijing injections can reduce the inflammatory response, thereby improving short-term cardiac function and promoting ECMO weaning in patients with acute myocardial infarction to a limited extent. However, larger scale multi-center trials are needed to confirm the reliability and effectiveness of Xuebijing.

## Availability of Data and Materials

The data are available from the corresponding author on reasonable request.
